# Overexpression of the pro‐protein convertase furin predicts prognosis and promotes papillary thyroid carcinoma progression and metastasis through RAF/MEK signaling

**DOI:** 10.1002/1878-0261.13396

**Published:** 2023-02-27

**Authors:** Pratheesh Kumar Poyil, Abdul K. Siraj, Divya Padmaja, Sandeep Kumar Parvathareddy, Roxanne Diaz, Saravanan Thangavel, Rafia Begum, Wael Haqawi, Falah Hassan Al‐Mohanna, Saif S. Al‐Sobhi, Fouad Al‐Dayel, Khawla S. Al‐Kuraya

**Affiliations:** ^1^ Human Cancer Genomic Research King Faisal Specialist Hospital and Research Center Riyadh Saudi Arabia; ^2^ Department of Comparative Medicine King Faisal Specialist Hospital and Research Centre Riyadh Saudi Arabia; ^3^ Department of Surgery King Faisal Specialist Hospital and Research Centre Riyadh Saudi Arabia; ^4^ Department of Pathology King Faisal Specialist Hospital and Research Centre Riyadh Saudi Arabia

**Keywords:** apoptosis, *BRAF*, EMT, Furin, PTC

## Abstract

Furin belongs to the pro‐protein convertases (PCs) family and its aberrant expression has been documented in various types of cancers; however, its role in thyroid cancer remains unclear. We investigated the expression of furin in a large cohort of Middle Eastern papillary thyroid carcinoma (PTC) patient samples and explored its functional role and mechanism in PTC cell lines *in vitro* and *in vivo*. Furin overexpression was observed in 44.6% of all PTC cases and was significantly associated with aggressive clinicopathological parameters and poor outcomes. We show that the knockdown of *FURIN* suppresses tumor growth, proliferation, migration, invasion, spheroid growth, and progression of epithelial‐to‐mesenchymal transition (EMT) in B‐Raf proto‐oncogene, serine/threonine kinase (*BRAF*) mutant cells, whereas its overexpression in *BRAF* wild‐type PTC cell lines reversed the effect. *FURIN* knockdown in the *BRAF* mutant cell line led to reduced tumor growth and increased apoptosis. Mechanistically, *FURIN* knockdown led to MEK/ERK pathway suppression in *BRAF* mutant cells, although inhibition of MEK did not affect furin expression, which suggests that furin acts through the MEK/ERK pathway. Furthermore, our study revealed the synergistic antitumor effect of furin depletion and anti‐MEK inhibitor treatment. Overall, these results indicate that furin is an important prognostic marker in Middle Eastern PTC and that it plays a crucial role in *BRAF*‐associated MAP/ERK pathway activation and tumorigenesis. Furin inhibition could be a potential therapeutic target for aggressive PTC.

## Graphical abstract

The authors show that furin is overexpressed in papillary thyroid carcinoma (PTC) and is significantly associated with aggressive clinicopathological parameters and poor outcomes. Functional studies show that furin promotes proliferation, tumor growth, migration, invasion, stemness and progression of EMT transition in PTC cell lines via the MEK/ERK pathway. Furin inhibition is a potential therapeutic target for a subset of PTC.

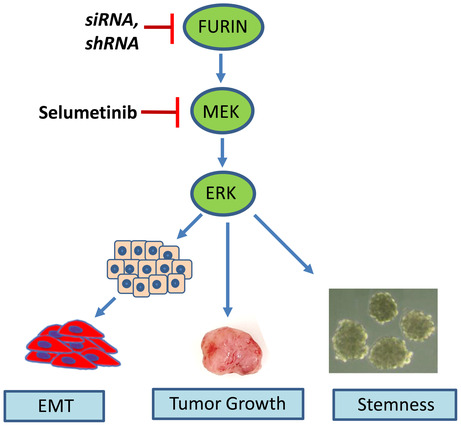

**Abbreviations**
ATCC: American‐type culture collection
DSMZ: Deutsche Sammlung von Mikroorganismen und Zellkulturen GmbH
EMT: epithelial to mesenchymal transition
ERK1/2: extracellular signal‐regulated protein kinase
FBS: fetal bovine serum
IHC: immunohistochemistry
PARP: Poly (ADP‐ribose) polymerase
PCR: polymerase chain reaction
PTC: papillary thyroid cancer
TMA: tissue microarray

## 1 Introduction

Papillary thyroid cancer is the most common endocrine malignancy. The incidence of PTC is on the rise worldwide [1, 2, 3]. In Saudi Arabia, PTC is the second commonest cancer affecting women after breast cancer [4]. Despite the indolent nature of PTC, there are considerable percentages (10–15%) of patients who presented with aggressive disease and develop resistance to standard therapeutic approaches [5, 6, 7, 8].

Constitutive activation of the mitogen‐activated protein kinase (MAPK) signaling pathway is a major event in the carcinogenesis of PTC [9]. Mutations in *BRAF* and *KRAS* genes are found in ~ 60% of PTC tumors [10]. Several studies have linked PTC's aggressive phenotype and poor clinical outcome to *BRAF* mutations [11, 12]. This highlights the importance of identifying a new therapeutic target that can interfere with this signaling pathway and inhibit tumor progression.

Furin, pro‐protein convertase subtilisin/kexin family member 3, is a calcium‐dependent protease and it is present in all mammalian cells [13]. Furin with the other six members (PC1/3, PC2, PACE 4, PC4, PC5/6, and PC7/8) constitute the pro‐protein convertases family that is responsible for cleavage and activation of various protein precursors in cells [14, 15]. The maturation of several of these precursors promotes many cancer‐related processes such as proliferation, migration and invasion, and metastasis [16, 17, 18, 19], which strongly indicate that furin could play an important role in tumorigenesis.

Numerous studies have reported the overexpression of furin in several cancers including sarcoma [14], hepatocellular carcinoma [20], breast cancer [21], head and neck [22], ovarian cancer [23], lung cancer [24], skin tumor [25], and colorectal cancer [26]. Interestingly, several studies have suggested that inhibition of furin can suppress the tumorigenic properties and metastatic potential of many cancers [18, 27, 28, 29, 30, 31]. Furthermore, previous studies have indicated that furin inhibition or silencing inhibits the processing of insulin growth factor‐1 receptor (IGF1R), matrix metalloproteinase 2 (MMP‐2) and leads to downregulation of PI3K/AKT and ERK–MAPK pathways [17, 26, 31, 32].

A recent study has shown that furin plays an important role in *BRAF*‐associated pathway activation in colorectal cancer [26]. This intrigued us to conduct this study to investigate for the first time whether the expression of furin in a large cohort of Middle Eastern PTC and if it plays any role in the activation of the *BRAF*‐MAPK signaling pathway in thyroid cancer.

## 2 Materials and methods

### 2.1 Patient selection

A total of 1479 patient samples diagnosed with primary PTC between 1988 and 2018 at King Faisal Specialist Hospital & Research Centre (Riyadh, Saudi Arabia) and Prince Sultan Military Medical City (Riyadh, Saudi Arabia) were collected from the Department of Pathology. Detailed clinicopathological data, including follow‐up data, were noted from case records and summarized in Table 1. In addition, distant metastatic tissue was available for 15 of these cases. Overall survival was defined as the length of time after primary treatment for cancer ends that the patient survives. The study was conducted according to the guidelines of the Declaration of Helsinki, and approved by the Institutional Review Board (IRB) and Research Ethics Committee (REC) of the King Faisal Specialist Hospital & Research Centre (KFSH&RC). Waiver of consent was obtained for all archived paraffin tissue blocks, including normal tissue blocks used as control, from IRB of the KFSH&RC under Research Advisory Council (RAC) on 07 February 2022 (RAC#2220002).

**Table 1:** Patient characteristics for adult PTC (*n* = 1430).

	Total	
	No.	%
Total		
Age (in years)		
Median (range)		
< 55	1208	81.7
≥ 55	271	18.3
Sex		
Female	1124	76.0
Male	355	24.0
Histologic subtype		
Classical variant	976	66.0
Follicular variant	261	17.6
Tall cell variant	133	9.0
Other variants	109	7.4
Extrathyroidal extension		
Present	629	42.5
Absent	850	57.5
Tumor focality		
Unifocal	741	50.1
Multifocal	738	49.9
Tumor laterality		
Unilateral	999	67.5
Bilateral	480	32.5
Lymphovascular invasion		
Present	316	21.4
Absent	1163	78.6
pT		
T1	575	38.9
T2	463	31.3
T3	285	19.3
T4	110	7.4
Unknown	46	3.1
pN		
N0	651	44.0
N1	732	49.5
Nx	96	6.5
pM		
M0	1402	94.8
M1	77	5.2
TNM stage		
I	1230	83.2
II	157	10.6
III	20	1.4
IV	50	3.4
Unknown	22	1.4
*BRAF* mutation		
Present	800	54.1
Absent	640	43.3
Unknown	39	2.6
*NRAS* mutation		
Present	101	6.8
Absent	1329	89.8
Unknown	49	3.3
*HRAS* mutation		
Present	33	2.2
Absent	1409	95.3
Unknown	37	2.5
*KRAS* mutation		
Present	8	0.5
Absent	1426	96.5
Unknown	45	3.0

### 2.2 DNA isolation

DNA was isolated from formalin‐fixed, paraffin‐embedded (FFPE) tumor tissues using a Gentra DNA isolation kit (Gentra, Minneapolis, MN, USA), following the manufacturer's recommendations as described previously [33].

### 2.3 PCR and sanger sequencing

primer 3 software was used to design the primers for entire coding and splicing regions of *MAPK* genes exons 1 and 2 in *HRAS*, *KRAS*, and *NRAS* and exon 15 in *BRAF* (primers available upon request). PCR was performed in a total volume of 25 μL with 20 ng of genomic DNA, 2.5 μL 10 × Taq buffer, 2.3 mm dNTPs, 1‐unit Taq polymerase, and 0.2 μm each primer and de‐ionized water. The efficiency and quality of the amplified PCR products were confirmed by loading them on a 2% agarose gel.

The PCR products were subsequently subjected to direct sequencing with BigDye terminator V 3.1 cycle sequencing reagents and analyzed on an ABI 3730XL DNA analyzer (Applied Biosystems, Foster City, CA, USA). Reference sequences were downloaded from NCBI GenBank. Sequencing traces were analyzed with the mutation surveyor v4.04 (Soft Genetics, LLC, State College, PA, USA) as described earlier [33].

### 2.4 Tissue microarray (TMA) construction and immunohistochemistry

All samples were analyzed in a tissue microarray (TMA) format. Tissue microarray construction was performed from formalin‐fixed, paraffin‐embedded PTC specimens as described previously [34, 35]. Briefly, Tissue cylinders with a diameter of 0.6 mm were punched from representative tumor regions of each donor tissue block and brought into the recipient paraffin block using a modified semiautomatic robotic precision instrument (Beecher Instruments, Woodland, WI, USA). Two cores of papillary thyroid carcinoma were arrayed from each case.

Standard protocol was followed for IHC staining. For antigen retrieval, Dako (Dako Denmark A/S, Glostrup, Denmark) Target Retrieval Solution pH 9.0 (Catalog number S2367) was used, and the slides were placed in a Pascal pressure cooker for 8 min at 120 °C. The slides were incubated with primary antibody against furin (ab183495, Abcam, Cambridge, UK) with a dilution of 1 : 100 (pH 9.0). The Dako Envision Plus System kit was used as the secondary detection system with 3, 3′‐diaminobenzidine as the chromogen. All slides were counterstained with hematoxylin, dehydrated, cleared, and mounted. Negative controls included the omission of the primary antibody. Normal tissues of different organs were also included in the TMA to serve as a control. Only fresh‐cut slides were stained simultaneously to minimize the influence of slide aging and maximize the reproducibility of the experiment.

H score was used to quantify furin protein expression based on intensity and proportion of staining, as described previously by us [36]. The intensity of staining was scored from 0 to 3 (0: absent, 1+: weak, 2+: moderate, 3+: strong), and the proportion of tumor cells staining for that particular intensity was recorded as 5% increments from a range of 0–100. A final *H* score was assigned using the following formula: *H* score = [1 × (% cells 1+) + 2 × (% cells 2+) + 3 × (% cells 3+)]. This *H* score ranges from 0 to 300. Two scores per tumor were analyzed to minimize the number of missing/un‐interpretable spots. However, for the final score, we used the higher of the two scores. X‐tile plots were constructed for the assessment of biomarkers and optimization of cutoff points based on outcome as has been described earlier [37]. Based on X‐tile plots, PTC cases were classified into two subgroups: those with an *H* score ≤ 75 were defined as low expression of furin, and those with an *H* score > 75 were defined as over‐expression.

### 2.5 Cell culture

The PTC cell line, BCPAP (RRID:CVCL_0153), was obtained from DSMZ, and TPC‐1 (RRID:CVCL_6298) was kindly provided by Dr Bryan McIver (Department of Endocrinology, Mayo Clinic, Rochester, Minnesota). The K1 (RRID:CVCL_2537) cell line was purchased from American Type Culture Collection (ATCC). Cell lines were cultured in RPMI 1640 media supplemented with 10% fetal bovine serum (FBS), 100 Units·mL^−1^ penicillin/streptomycin, and 100 Units·mL^−1^ Glutamine. These cell lines were tested negative for mycoplasma, and cell line authentication was performed in‐house via short tandem repeat DNA fingerprinting using the AmpFISTR identifier PCR Amplification Kit (Thermo Fisher, Waltham, MA, USA) and the results were in concordance with published data [38, 39]. All experiments were performed using 5% FBS in RPMI 1640 media.

### 2.6 Reagents and antibodies

Antibodies against Furin (ab183495), N‐cadherin (ab98952), and Twist (ab175430) were purchased from Abcam Inc (Cambridge, MA, USA). Antibodies against pMEK1/2 (9121), MEK1/2 (4694), pERK1/2 (4370), ERK1/2 (4695), PARP (9542), β‐Actin (3700), E‐cadherin (3195), Nanog (4903), CD44 (3570) and CD133 (64326) were purchased from Cell Signaling Technology (Danvers, MA, USA). Zeb1 antibody (NBP1‐05987) was purchased from Novus Biologicals (Minneapolis, MN, USA). Caspase‐3 (sc‐56053) antibody was purchased from Santa Cruz Biotechnology, Inc. (Santa Cruz, CA, USA). Selumetinib (AZD6244) was purchased from Selleck chemicals (Houston, TX, USA) and Mirdametinib (PD0325901) was purchased from Cayman Chemicals (Ann Arbor, MI, USA). The zVAD‐fmk was purchased from Calbiochem (San Diego, CA, USA).

### 2.7 Clonogenic assay

The single‐cell suspensions of exponentially growing PTC cell lines were seeded into a six‐well plate at a low density of 500 cells per well and allowed to adhere. After attachment, the cell culture medium was refreshed and cells were allowed to grow for 8–10 days. Cell colonies were fixed with formaldehyde (4%) and stained with crystal violet (2% in 10% methanol). The number of colonies in each well was counted and photographed.

### 2.8 Gene silencing using siRNA

Cells were transfected with two different sequences of *FURIN* siRNA's SR321213A‐5'r(AUAGUUGAGCCCCAAGUCCUGAAGA)3′ and SR321213B‐5'r(AACAGUAUCUA CACGCUGUCCAUCA)3′ and scrambled negative control siRNA (SR30004) from OriGene (Rockville, MD, USA) using Lipofectamine 2000 (Invitrogen, Carlsbad, CA, USA) for 6 h following which the lipid and siRNA complex was removed and fresh growth medium was added. After 48 h of transfection, cells were used for immunoblotting.

### 2.9 Plasmid and transfection

*FURIN* cDNA encoding human *FURIN* (RC204279) and shRNA's (TR312903A: AAAGTGATTAAACGTGCAGACTATGCAAA and TR312903B: ACGGCATTGTGGTCTCCATTCTGGACGAT) targeting human *FURIN* were purchased from Origene. The overexpression of *FURIN* and knockdown of *FURIN* in PTC cells were performed using Lipofectamine™2000 (Invitrogen) according to the manufacturer's protocol. Briefly, PTC cells were seeded in 6‐well culture plates; when approximately 50% confluent, cells were transfected with 4 μg plasmid for 48 h. Stable overexpression clones resistant to G418 and stable knockdown clones resistant to puromycin were isolated; overexpression and knockdown of furin protein production were confirmed by immunoblotting.

### 2.10 Cell invasion and migration assays

Cell invasion and migration assays were performed as described previously [38]. Briefly, cells, after treatment were seeded into trans‐well, inserts either uncoated (for migration assay) or coated (for invasion assay) with growth factor‐reduced matrigel for 24 h. After incubation, cells were stained with a Diff‐Quick stain set (Fisher Scientific, Pittsburg, PA, USA), and photographed under a fluorescent microscope.

### 2.11 Sphere forming assay

Sphere forming assay was performed as described previously [40]. Briefly, PTC cells (500 per well) were plated on Corning 24‐well ultra‐low attachment plates (Corning Inc., Kennebunk, ME, USA) grown in serum‐free DMEM‐F12 (ATCC) supplemented with B27 (Gibco, Grand Island, NY, USA), 20 ng·mL^−1^ epidermal growth factor (Sigma‐Aldrich, Saint Louis, MO, USA), 0.4% bovine serum albumin (Sigma‐Aldrich, Milwaukee, WI, USA) and 4 μg·mL^−1^ insulin (Sigma‐Aldrich, Saint Louis, MO, USA). Fresh medium was supplemented every 2 days. The spheroids were counted and photographed on day 14. For secondary spheroid formation, the primary spheroids were dissociated into single cells, and cultured on 24‐well ultra‐low attachment plates using a spheroid culture medium for another 10 days.

### 2.12 Animals and xenografts study

Six‐week‐old female nude mice (NU/J) were obtained from Jackson Laboratories (Bar Harbor, ME, USA) and maintained in a sterile and pathogen‐free environment (12 h light/12 h darkness) with food and water *ad libitum* at least 1 week before use. All animal studies were done in accordance with institutional guidelines as described previously [41] and the study was approved by the animal use and care committee (ACUC) of King Faisal Specialist Hospital and Research Center on 07 February 2022 (Approval No: RAC#2220002).

For the xenograft study, PTC cells (5 × 10^6^ cells per mouse) were re‐suspended in serum‐free medium with matrigel basement membrane matrix (BD Biosciences, Bedford, MA, USA) at a 1 : 1 ratio (total volume = 100 μL) and subcutaneously injected into the flanks of NU/J mice (*n* = 5). After tumors grew to about 100 mm^3^, mice were treated intraperitoneally with selumetinib (10 mg·kg^−1^) and vehicle (0.1% DMSO), twice a week for 30 days. The body weight and tumor volume of each mouse were monitored weekly. After 4 weeks of treatment, mice were sacrificed and individual tumors were weighed, then snap‐frozen in liquid nitrogen for storage.

### 2.13 Statistical analysis

Contingency table analysis and chi‐square tests were used to study the relationship between clinicopathological variables and furin expression. Survival curves were generated using the Kaplan–Meier method, with significance evaluated using the Mantel‐Cox log‐rank test. Cox proportional hazards regression model was used for multivariate analysis. The limit of significance for all analyses was defined as a *P*‐value of 0.05; two‐sided tests were used in all calculations. The jmp11.0 (SAS Institute, Inc., Cary, NC, USA) software package was used for data analyses.

For all functional studies, data presented are means ± SD of triplicates in an independent experiment, which was repeated at least two times with the same results. Student *t*‐test (two‐tailed) was performed for statistical significance with a *P* < 0.05 used as the cut‐off.

## 3 Results

### 3.1 Furin expression in normal thyroid, primary tumor, and distant metastatic tumor tissues

**Fig. 1:**
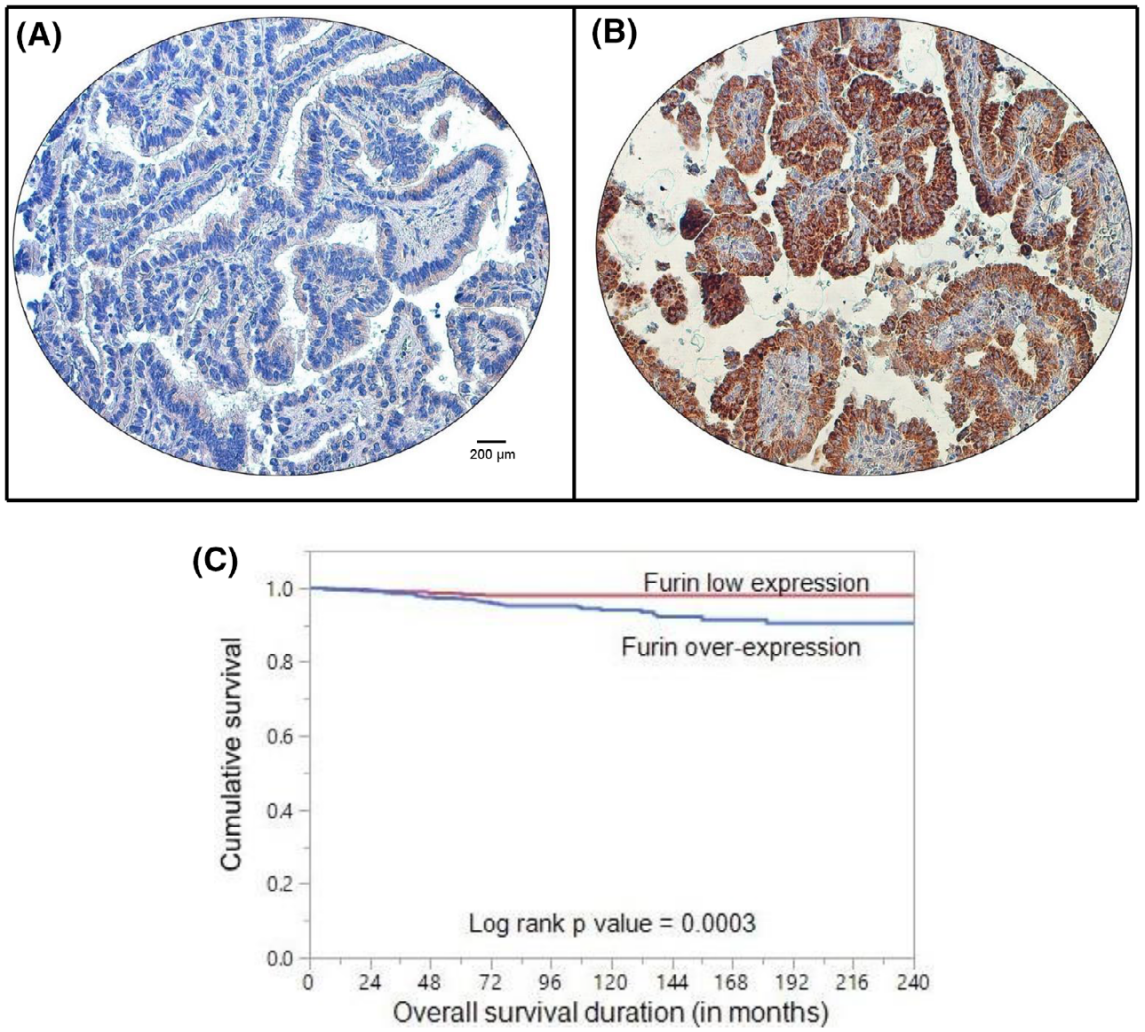
Immunohistochemical analysis of furin expression in papillary thyroid cancer (PTC) TMA. Representative examples of tumors showing (A) low expression and (B) high expression of furin. (20×/0.70 objectives on an Olympus BX 51 microscope (Olympus America Inc, Center Valley, PA, USA) with the inset showing a 40× 0.85 aperture magnified view of the same TMA spot; scale bar in A and B = 200 μm). (C) Kaplan–Meier survival analysis for the prognostic significance of furin expression in PTC. PTC patients with overexpression of furin had reduced disease‐free survival at 5 years compared to tumors showing low expression of Furin (*P* = 0.0003).

Furin was primarily expressed in the cytoplasm (Fig. 1A,B). Furin over‐expression was noted in 44.6% (659/1479) of primary PTCs compared to 5.8% (14/242) of normal thyroid tissues and the difference was statistically significant (*P* < 0.0001). We also analyzed the expression of furin in 15 distant metastatic tissues and found that furin was overexpressed in 73.3% (11/15) of metastatic tissues, which was significantly higher than that noted in primary PTC (*P* = 0.0262).

### 3.2 Association of furin expression with clinicopathological factors and outcome in papillary thyroid carcinoma

Furin overexpression was significantly associated with adverse clinicopathological parameters such as older age (*P* < 0.0001), tall cell variant (*P* < 0.0001), extrathyroidal extension (*P* < 0.0001), T4 tumors (*P* = 0.0004), advanced stage (*P* < 0.0001) and proliferation marker Ki‐67 (*P* = 0.0007). Interestingly, we also found a significant association between furin over‐expression and *BRAF* mutation (*P* < 0.0001) in our cohort (Table 2).

**Table 2:** Association of clinico‐pathological characteristics with Furin expression in PTC.

	Total		Furin expression				*P* value
			High		Low		
	No.	%	No.	%	No.	%	
No. of patients	1479		659	44.6	820	55.4	
Age (in years)							
< 55	1208	81.7	490	74.4	718	87.6	< 0.0001
≥ 55	271	18.3	169	25.6	102	12.4	
Sex							
Female	1124	76.0	507	76.9	617	75.2	0.4488
Male	355	24.0	152	23.1	203	24.8	
Histology subtype							
Classical variant	976	66.0	422	64.0	554	67.6	< 0.0001
Follicular variant	261	17.6	81	12.3	180	21.9	
Tall cell variant	133	9.0	101	15.3	32	3.9	
Other variants	109	7.4	55	8.4	54	6.6	
Extrathyroidal extension							
Present	629	42.5	321	48.7	308	37.6	< 0.0001
Absent	850	57.5	338	51.3	512	62.4	
Tumor focality							
Unifocal	741	50.1	343	52.0	398	48.5	0.1794
Multifocal	738	49.9	316	48.0	422	51.5	
Tumor laterality							
Unilateral	999	67.5	456	69.2	543	66.2	0.2239
Bilateral	480	32.5	203	30.8	277	33.8	
Lymphovascular invasion							
Present	316	21.4	147	22.3	169	20.6	0.4292
Absent	1163	78.6	512	77.7	651	79.4	
pT							
pT1	575	40.1	246	38.5	329	41.4	0.0004
pT2	463	32.3	195	30.5	268	33.8	
pT3	285	19.9	128	20.0	157	19.8	
pT4	110	7.7	70	11.0	40	5.0	
pN							
pN0	651	47.1	272	44.2	379	49.4	0.0514
pN1	732	52.9	344	55.8	388	50.6	
pM							
pM0	1402	94.8	629	95.4	773	94.3	0.3078
pM1	77	5.2	30	4.6	47	5.7	
Stage							
I	1230	84.4	512	79.3	718	88.5	< 0.0001
II	157	10.8	85	13.2	72	8.9	
III	20	1.4	16	2.5	4	0.5	
IV	50	3.4	33	5.1	17	2.1	
Ki‐67 expression							
Present	251	17.4	136	21.1	115	14.3	0.0007
Absent	1195	82.6	508	78.9	687	85.7	
*BRAF* mutation							
Present	800	55.6	431	67.2	369	46.2	< 0.0001
Absent	640	44.4	210	32.8	430	53.8	
*NRAS* mutation							
Present	101	7.1	37	5.8	64	8.1	0.0862
Absent	1329	92.9	603	94.2	726	91.9	
*HRAS* mutation							
Present	33	2.3	15	2.3	18	2.3	0.9132
Absent	1409	97.7	627	97.7	782	97.7	
*KRAS* mutation							
Present	8	0.6	3	0.5	5	0.6	0.6849
Absent	1426	99.4	636	99.5	790	99.4	

We also found a significant association between furin expression and overall survival (*P* = 0.0003; Fig. 1C), which remained significant even after adjusting for other clinicopathological parameters on multivariate Cox proportional hazards model (Hazard ratio = 2.43; 95% confidence interval = 1.09–6.01; *P*‐value = 0.0298; Table 3), thus establishing furin as an independent predictor of overall survival in PTC for this cohort.

**Table 3:** Multivariate analysis of overall survival using Cox Proportional Hazard Model.

Clinico‐pathological variables	Overall survival		
	Hazard ratio	95% confidence interval	*P*‐value
Age Above ≥ 55 years (vs. < 55 years)	10.41	4.19–31.55	< 0.0001
Sex Male (vs. Female)	1.38	0.63–2.86	0.4058
Histology Tall cell variant (vs. other variants)	2.71	1.16–5.93	0.0220
Extrathyroidal extension Present (vs. Absent)	0.86	0.31–2.56	0.7815
pT T3 & T4 (vs. T1 & T2)	1.68	0.57–5.08	0.3471
Lymph node metastasis N1 (vs. N0)	1.62	0.76–3.69	0.2183
Distant metastasis Present (vs. absent)	3.76	1.08–12.30	0.0376
Stage III‐IV (vs. I–II)	0.92	0.26–2.83	0.8939
*BRAF* mutation Present (vs. Absent)	0.91	0.41–2.19	0.8347
Furin expression High (vs. Low)	2.43	1.09–6.01	0.0298

### 3.3 Furin promotes PTC cell growth *in vitro*

**Fig. 2:**
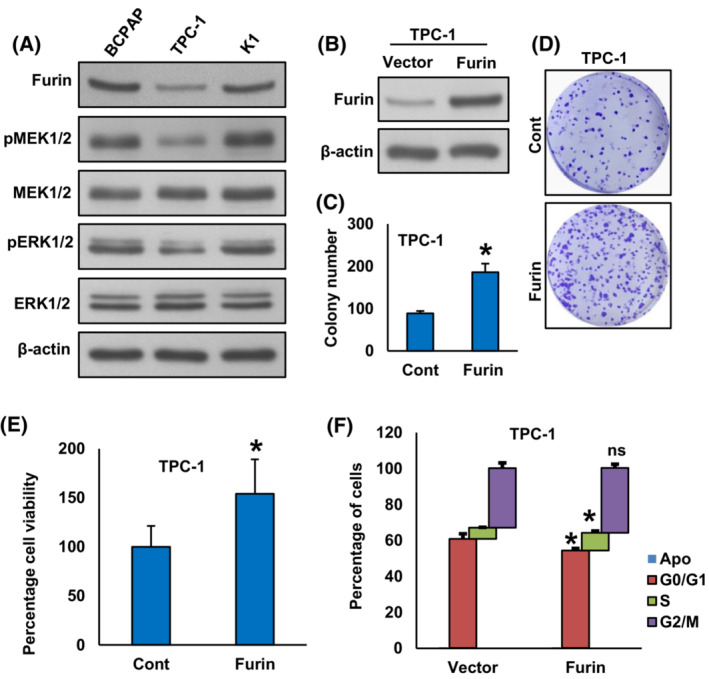
Furin promotes PTC cell growth *in vitro*. (A) Basal expression of furin in PTC cell lines. Proteins were isolated from three PTC cell lines and immunoblotted with antibodies against Furin, pMEK1/2, MEK1/2, pERK1/2, ERK1/2 and β‐Actin (*n* = 3). (B) Forced expression of furin in low‐expressing cells. TPC‐1 cells were transfected with either an empty vector or *FURIN* cDNA and overexpression was confirmed by immunoblotting (*n* = 3). (C, D) Forced expression of furin increases clonogenicity. TPC‐1 cells were transfected with either an empty vector or *FURIN* cDNA for 48 h. Selected clones were seeded at a density of 500 cells per well in a 6‐well plate, and grew for an additional 10 days, then stained with crystal violet, and colonies were counted. Data were presented as mean ± SD (*n* = 3). (E) Furin promotes TPC‐1 cell proliferation. TPC‐1 cells carrying empty vector and *FURIN* cDNA were grown for 48 h and cell proliferation was measured by MTT assay. Data were presented as mean ± SD (*n* = 6). (F) TPC‐1 cells were transfected with either an empty vector or *FURIN* cDNA for 48 h. Cells were stained with propidium iodide (PI) followed by flow cytometry analysis. Data were presented as mean ± SD (*n* = 3). Statistical analyses were performed using two‐tailed Student's *t*‐tests. *P < 0.05.

Our clinical data showed furin overexpression in PTC cases and was significantly associated with *BRAF* mutation. To test this association and function of furin *in vitro*, we first analyzed the basal expression of furin in three PTC cell lines by immunoblotting. As shown in Fig. 2A, we found high expression of furin along with pMEK1/2 and pERK1/2 in *BRAF* mutated PTC cell lines (BCPAP and K1) compared to *BRAF* wild‐type PTC cell line (TPC‐1). Next, we overexpressed furin in the TPC‐1 cell line (Fig. 2B) and evaluated the cell growth by clonogenic assay. As shown in Fig. 2C,D, forced expression of furin significantly increased cell growth. Furthermore, ectopic expression of furin significantly increased the cell proliferation after 48 h of seeding as measured by MTT assay (Fig. 2E) and comparative viable cell counting analysis using Bio‐Rad TC20 automated cell counter (data not shown).

In addition, we investigated whether the overexpression of furin affects the distribution of cells in the different cell cycle phases (Fig. 2F). We observed a significant increase in the percentage of cells in the S phase, the most proliferative phase, from 6.1 ± 0.2% in empty vector‐transfected TPC‐1 cells to 9.9 ± 0.9% in furin overexpressing TPC‐1 cells. At the same time, cells that overexpress furin showed significantly fewer cells in the G0/G1 phase of the cell cycle (Fig. 2F). These results indicate that the upregulation of furin increases the cell proliferation ability of PTC cells.

**Fig. 3:**
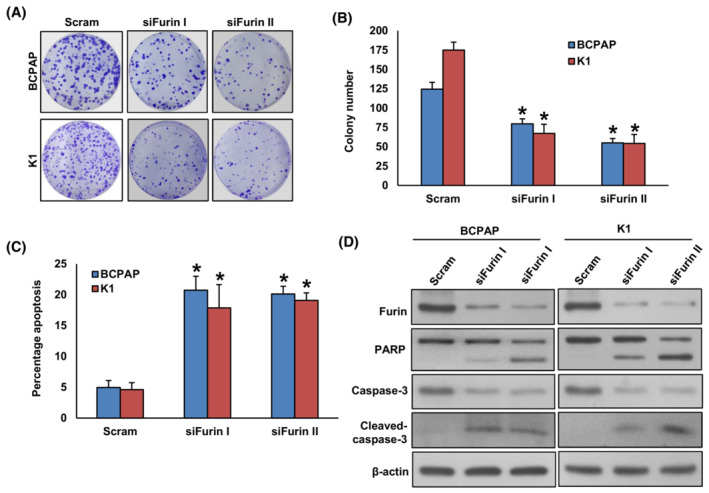
Knockdown of *FURIN* decrease PTC cell growth and induce apoptosis. (A, B) Silencing of *FURIN* attenuates clonogenicity. PTC cells were transfected with scrambled siRNA and two different *FURIN* siRNA sequences (25 nm). After 48 h, cells were seeded at a density of 500 cells per well in a 6‐well plate and grown for an additional 10 days, then stained with crystal violet, and colonies were counted. Data were presented as mean ± SD (*n* = 3). (C) Depletion of furin induces apoptosis in PTC cell lines. After 48 h of transfection, cells were stained with fluorescein‐conjugated annexin‐V and propidium iodide (PI) and analyzed by flow cytometry. Data were presented as mean ± SD (*n* = 3). (D) Depletion of furin induces the cleavage of caspase‐3 and PARP. After 48 h of transfection, cells were lysed and proteins were immuno‐blotted with antibodies against Furin, PARP, casapse‐3, cleaved casapse‐3, and β‐Actin as indicated (*n* = 3). Statistical analyses were performed using two‐tailed Student's *t*‐tests. *P < 0.05.

On the contrary, the knockdown of *FURIN* in BCPAP and K1 significantly decreased cell growth (Fig. 3A,B) and induced apoptosis (Fig. 3C). We also investigated the expression of apoptotic protein markers after *FURIN* knockdown on these cells by immunoblotting. As shown in Fig. 3D, the knockdown of *FURIN* also induced the cleavage of PARP and caspase‐3 in these cells. To confirm whether the decrease in cell growth after furin depletion was due to apoptosis, PTC cells were pre‐treated with a universal inhibitor of apoptosis, zVAD‐fmk (80 μm) for 3 h followed by transfection with *FURIN* siRNA for 48 h. As shown in Fig. S1A, there was a significant inhibition of apoptosis in zVAD‐fmk pre‐treated cells as compared to *FURIN* siRNA transfected cells alone. Furthermore, zVAD‐fmk pre‐treatment appreciably improved the cell growth in *FURIN* knockdown cells (Fig. S1B).

To identify whether inhibition of cell growth after *FURIN* knockdown was associated with cell cycle arrest, we performed cell cycle analysis on PTC cell lines following transfection with two separate *FURIN* siRNA sequences for 48 h (Fig. S1C). There was a significant increase in the sub‐G1 (Apo) fraction of cells in both *FURIN* siRNA transfected PTC cell lines compared to scramble siRNA transfected controls suggesting that these cells were dying of apoptosis (Fig. S1C).

### 3.4 Furin activates MEK/ERK pathway

**Fig. 4:**
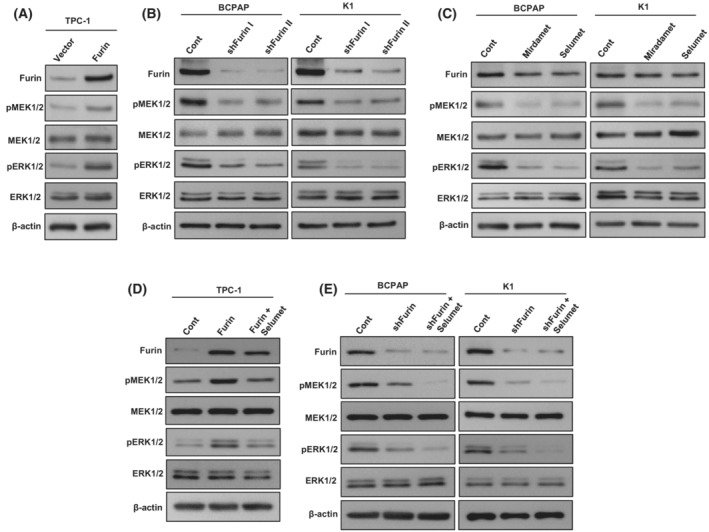
Furin activates MEK/ERK pathway. (A) Forced expression of furin stimulates MEK/ERK pathway. TPC‐1 cells were transfected with either an empty vector or *FURIN* cDNA for 48 h. Cells were lysed and proteins were immuno‐blotted with antibodies against Furin, pMEK1/2, MEK1/2, pERK1/2, ERK1/2, and β‐Actin as indicated (*n* = 3). (B) Depletion of furin inhibits MEK/ERK activation. PTC cells were transfected with a control shRNA vector or two different *FURIN* shRNA constructs. After transfection, cells were lysed and proteins were immuno‐blotted with antibodies against Furin, pMEK1/2, MEK1/2, pERK1/2, ERK1/2, and β‐Actin as indicated (*n* = 3). (C) The effect of MEK inhibitors on furin expression. PTC cell lines were treated with a pharmacological inhibitor for MEK, mirdametinib (10 nM), and selumetinib (20 nM) for 48 h and analyzed the expression of Furin, pMEK1/2, MEK1/2, pERK1/2, ERK1/2 by immunoblotting (*n* = 3). (D) Selumetinib downregulates MEK/ERK signaling cascade in furin‐expressing cells. TPC‐1 cells carrying *FURIN* cDNA were treated with selumetinib (20 nM) for 48 h. Cells were lysed and proteins were immuno‐blotted with antibodies against Furin, pMEK1/2, MEK1/2, pERK1/2, ERK1/2, and β‐Actin as indicated (*n* = 3). (E) The co‐inhibition of MEK and furin further downregulate MEK/ERK signaling cascade in PTC cells. PTC cells were transfected with *FURIN* shRNA, and treated with selumetinib (20 nM) for 48 h. Cells were lysed and proteins were immuno‐blotted with antibodies against Furin, pMEK1/2, MEK1/2, pERK1/2, ERK1/2, and β‐Actin as indicated (*n* = 3).

In an attempt to investigate the mechanism of furin‐induced cell growth, we overexpressed furin in the TPC‐1 cell line and analyzed the expression of MEK/ERK pathway proteins. As shown in Fig. 4A, forced expression of furin markedly increased the phosphorylation of MEK1/2 and ERK1/2 in the TPC‐1 cell line. However, stable knockdown of *FURIN* noticeably decreased the phosphorylation of MEK1/2 and ERK1/2 in BCPAP and K1 cell lines (Fig. 4B). To test whether MEK inhibition has any effect on furin expression, *BRAF* mutated cell lines were treated with pharmacological inhibitors for MEK, mirdametinib (10 nM), and selumetinib (20 nM) for 48 h and analyzed the expression of furin, pMEK1/2, and pERK1/2 by immunoblotting. As shown in Fig. 4C, inhibition of MEK by both mirdametinib and selumetinib prominently downregulated the expression of pMEK1/2 and pERK1/2 but did not affect furin expression. We also show that selumetinib treatment downregulated the phosphorylation of MEK1/2 and ERK1/2 in both furin expressing (Fig. 4D) and knockdown (Fig. 4E) cells with no further change in the furin expression. All these data indicate that furin acts through MEK/ERK signaling cascade.

**Fig. 5:**
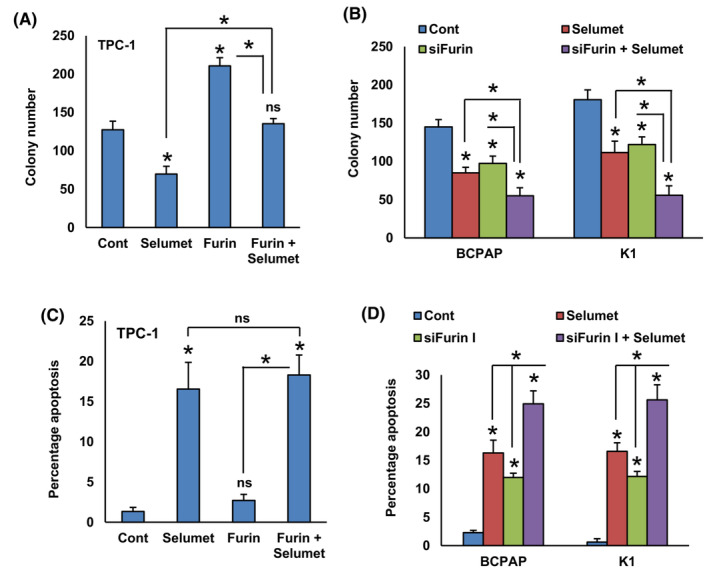
The MEK/ERK signaling pathway plays an important role in furin‐mediated cell growth and apoptosis. (A) TPC‐1 cells carrying empty vector or *FURIN* cDNA were treated with selumetinib (20 nM) for 48 h and the cells were subjected to clonogenic assay. Data were presented as mean ± SD (*n* = 3). (B) PTC cells were transfected with *FURIN* siRNA and treated with selumetinib (20 nM) for 48 h and the cells were subjected to clonogenic assay. Data were presented as mean ± SD (*n* = 3). (C) TPC‐1 cells carrying empty vector or *FURIN* cDNA were treated with selumetinib (20 nM). After 48 h, cells were analyzed for apoptosis by annexin‐V/PI. Data were presented as mean ± SD (*n* = 3). (D) PTC cells were transfected with *FURIN* siRNA and treated with selumetinib (20 nM). After 48 h, cells were analyzed for apoptosis. Data were presented as mean ± SD (*n* = 3). Statistical analyses were performed using two‐tailed Student's *t*‐tests. *P < 0.05.

To test the role of the MEK/ERK signaling pathway in furin‐mediated cell growth, we treated selumetinib in both furin‐expressing and knockdown cells and performed clonogenic assay and annexin V/PI dual staining for apoptosis. As expected, inhibition of MEK by selumetinib significantly decreased cell growth (Fig. 5A,B) and induced apoptosis (Fig. 5C,D) in both furin expressing and knockdown cells. These data demonstrate the important role of the MEK/ERK signaling pathway in furin‐mediated cell growth and apoptosis.

### 3.5 Furin promotes cancer cell stemness in PTC cells

**Fig. 6:**
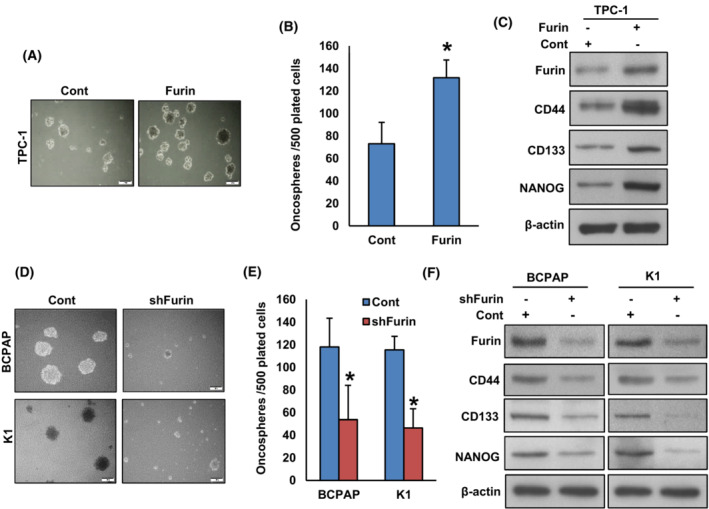
Furin promotes the self‐renewal ability of spheroids generated from PTC cells. (A, B) Forced expression of furin increases spheroid growth. TPC‐1 cells were transfected with either empty vector or *FURIN* cDNA and cells were subjected to sphere‐forming assay, (scale bar = 1 mm). Spheroids in the entire dish were counted. Data were presented as mean ± SD (*n* = 3). (C) Forced expression of furin increases the stemness of spheroids as confirmed by immunoblotting using stem cell markers. TPC‐1 cells were transfected with either empty vector or *FURIN* cDNA and grown in a sphere medium. Proteins were isolated from spheroids and immunoblotted with antibodies against Furin, CD44, CD133, NANOG, and β‐Actin (*n* = 3). (D, E) Depletion of furin attenuates the self‐renewal ability of spheroids. PTC cells were transfected with *FURIN* shRNA and cells were subjected to a sphere‐forming assay, (scale bar = 1 mm). Spheroids in the entire well were counted. Data were presented as mean ± SD (*n* = 3). (F) Depletion of furin reduces the stemness of spheroids. PTC cells were transfected with *FURIN* shRNA and grown in a sphere medium. Proteins were isolated from spheroids and immunoblotted with antibodies against Furin, CD44, CD133, NANOG, and β‐Actin (*n* = 3). Statistical analyses were performed using two‐tailed Student's *t*‐tests. *P < 0.05.

**Fig. 7:**
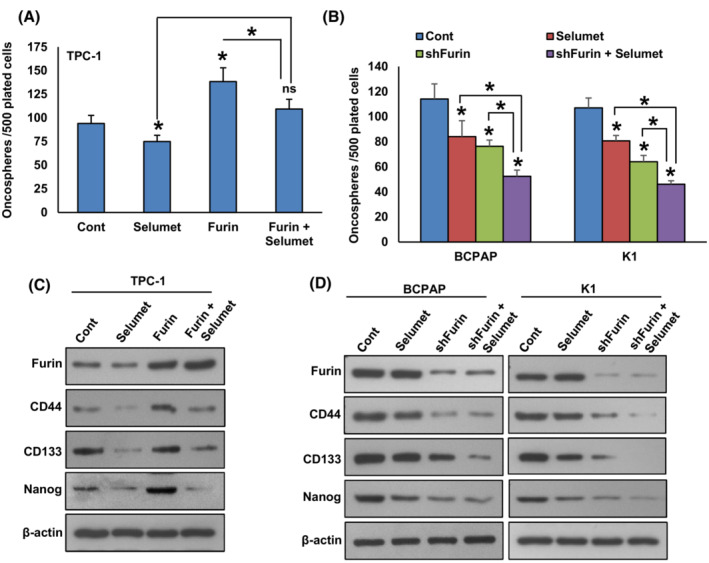
Inhibition of MEK decreases spheroid growth and stemness in both furin expressing and knockdown cells. (A) TPC‐1 cells carrying either empty vector or *FURIN* cDNA were treated with selumetinib (20 nM) for 48 h and the cells were subjected to sphere‐forming assay. Data were presented as mean ± SD (*n* = 3). (B) PTC cells carrying either empty vector or *FURIN* shRNA were treated with selumetinib (20 nM) for 48 h and the cells were subjected to sphere‐forming assay. Data were presented as mean ± SD (*n* = 3). (C) TPC‐1 cells carrying either empty vector or *FURIN* cDNA were treated with selumetinib (20 nM) for 48 h and grown in a sphere medium. Proteins were isolated from spheroids and immunoblotted with antibodies against Furin, CD44, CD133, NANOG, and β‐Actin (*n* = 3). (D) PTC cells carrying either empty vector or *FURIN* shRNA were treated with selumetinib (20 nM) for 48 h and grown in a sphere medium. Proteins were isolated from spheroids and immunoblotted with antibodies against Furin, CD44, CD133, NANOG, and β‐Actin (*n* = 3). Statistical analyses were performed using two‐tailed Student's *t*‐tests. *P < 0.05.

Furin has been associated with the stemness of colon cancer stem cells [42]. However, the role of furin in PTC stemness has not yet been investigated. To study the role of furin in spheroid growth in PTC, we overexpressed furin in TPC‐1 cells and were cultured in a spheroid medium. Forced expression of furin significantly increased the growth of spheroids generated from TPC‐1 cells (Fig. 6A,B). Also, furin overexpression elevated the levels of stem cell markers like CD44, CD133, and NANOG as compared to empty vector‐transfected cells (Fig. 6C). To validate the above findings, we stably silenced *FURIN* in BCPAP and K1 cell lines and grew them in a spheroid medium. As shown in Fig. 6D,E, the depletion of furin significantly reduced the spheroid growth in these cells and decreased the expression of stem cell markers (Fig. 6F). These results reveal the role of furin in maintaining cancer stemness properties in PTC cells. We also show that pharmacological inhibition of MEK by selumetinib significantly decreased spheroid growth (Fig. 7A,B) and stemness (Fig. 7C,D) in both furin expressing and knockdown cells, showing the role of MEK/ERK signaling pathway in furin mediated cancer stemness maintenance.

### 3.6 Furin activates EMT and metastatic potential in PTC cells

**Fig. 8:**
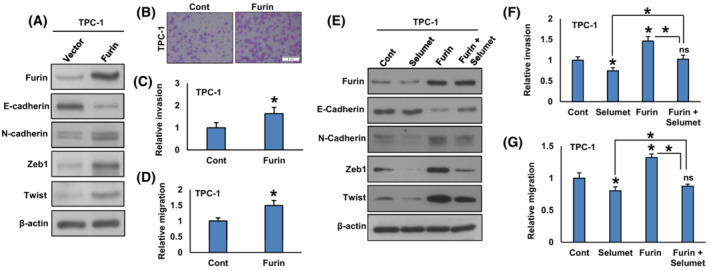
Forced expression of furin promotes EMT and metastatic potential of PTC cells. (A) TPC‐1 cells were transfected with either an empty vector or *FURIN* cDNA. After cell lysis, proteins were immuno‐blotted with antibodies against Furin, E‐cadherin, N‐cadherin, Zeb1, Twist, and β‐Actin as indicated (*n* = 3). (B, C) Forced expression of furin increases invasion. TPC‐1 cells were transfected with either an empty vector or *FURIN* cDNA. Cells were seeded into the upper compartment of invasion chambers. The bottom chambers were filled with RPMI media. After 24 h incubation, invaded cells were fixed, stained, and quantified (scale bar = 1 mm). Data were presented as mean ± SD (*n* = 3). (D) Forced expression of furin increases migration. After transfection, cells were seeded into the upper compartment of migration chambers. The bottom chambers were filled with RPMI media. After 24 h incubation, migrated cells were fixed, stained, and quantified. Data were presented as mean ± SD (*n* = 3). (E) Inhibition of MEK reduces EMT in furin‐expressing cells. TPC‐1 cells carrying either empty vector or *FURIN* cDNA were treated with selumetinib (20 nM) for 48 h. After cell lysis, proteins were immuno‐blotted with antibodies against Furin, E‐cadherin, N‐cadherin, Zeb1, Twist, and β‐Actin as indicated (*n* = 3). (F, G) Selumetinib decreases the invasion and migration of Furin expressing cells. TPC‐1 cells carrying either empty vector or *FURIN* cDNA were treated with selumetinib (20 nM) for 48 h and the cells were subjected to invasion and migration assay. Data were presented as mean ± SD (*n* = 3). Statistical analyses were performed using two‐tailed Student's *t*‐tests. *P < 0.05.

It has been reported that furin promotes EMT and its inhibition attenuates metastatic potential in pancreatic cancer cells [19]. The downregulation of furin inhibits migration and invasion in lung cancer [43] and rhabdomyosarcoma [44] cells. Therefore, we wanted to determine whether furin has any effect on inducing EMT and metastatic potential in PTC cell lines. We found that forced expression of furin increased the expression of Furin, N‐cadherin, Zeb1, and Twist with an associated downregulation of E‐cadherin expression in TPC‐1 cells (Fig. 8A). In addition, forced expression of furin also increased invasion (Fig. 8B,C) and migration (Fig. 8D) in TPC‐1 cells. In addition, treatment with selumetinib downregulated the expression of N‐cadherin, Zeb1, and Twist with a concomitant upregulation of E‐cadherin expression in furin‐expressing cells (Fig. 8E). Inhibition of MEK by selumetinib also significantly decreased invasion (Fig. 8F) and migration (Fig. 8G) of these cells.

**Fig. 9:**
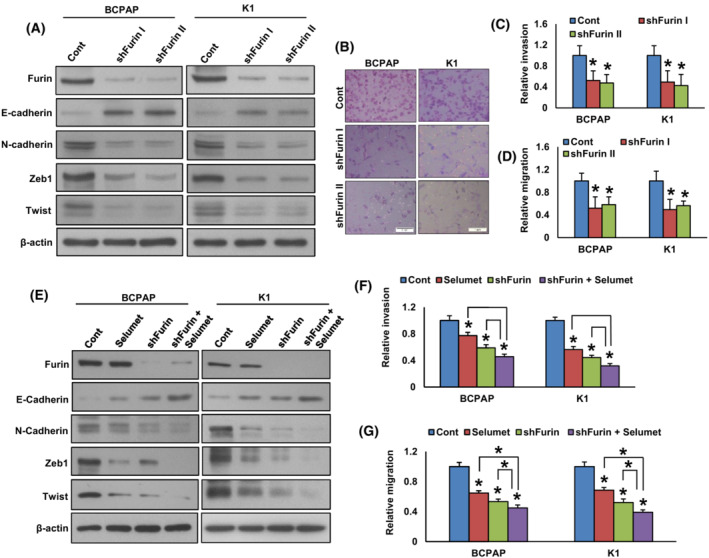
Depletion of furin attenuates EMT and metastatic potential of PTC cells. (A) PTC cells were transfected with a control shRNA vector or two different *FURIN* shRNA constructs. After transfection, cells were lysed and proteins were immuno‐blotted with antibodies against Furin, E‐cadherin, N‐cadherin, Zeb1, Twist, and β‐Actin as indicated (*n* = 3). (B, C) Knockdown of *FURIN* decreases the invasive ability of PTC cells. PTC cells were transfected with *FURIN* shRNA's and cells were subjected to invasion assay. (scale bar = 1 mm). Data were presented as mean ± SD (*n* = 3). (D) Knockdown of *FURIN* decreases the migratory ability of PTC cells. PTC cells were transfected with *FURIN* shRNA's and cells were subjected to migration assay. Data were presented as mean ± SD (*n* = 3). (E) The co‐inhibition of MEK and furin further reduce EMT in PTC cells. PTC cells carrying either empty vector or *FURIN* shRNA were treated with selumetinib (20 nM) for 48 h. After cell lysis, proteins were immuno‐blotted with antibodies against Furin, E‐cadherin, N‐cadherin, Zeb1, Twist, and β‐Actin as indicated (*n* = 3). (F, G) The co‐inhibition of MEK and furin further decreases the invasion and migration of PTC cells. PTC cells carrying either empty vector or *FURIN* shRNA were treated with selumetinib (20 nM) for 48 h and the cells were subjected to invasion and migration assay. Data were presented as mean ± SD (*n* = 3). Statistical analyses were performed using two‐tailed Student's *t*‐tests. *P < 0.05.

On the contrary, silencing of furin downregulated the expression of Furin, N‐cadherin, Zeb1, and Twist with a concomitant upregulation of E‐cadherin expression (Fig. 9A) in BCPAP and K1 cell lines. Depletion of furin also decreased invasion (Fig. 9B,C) and migration (Fig. 9D) of these cells. We show that inhibition of MEK along with furin further reduced the expression of N‐cadherin, Zeb1, and Twist with an increased E‐cadherin expression (Fig. 9E). Furthermore, co‐inhibition of MEK and furin significantly decreased invasion (Fig. 9F) and migration (Fig. 9G) of these cells. All these results demonstrate that furin promotes EMT and metastatic potential in PTC cells, where MEK/ERK signaling plays an important role.

### 3.7 Furin promotes tumor growth *in vivo*

**Fig. 10:**
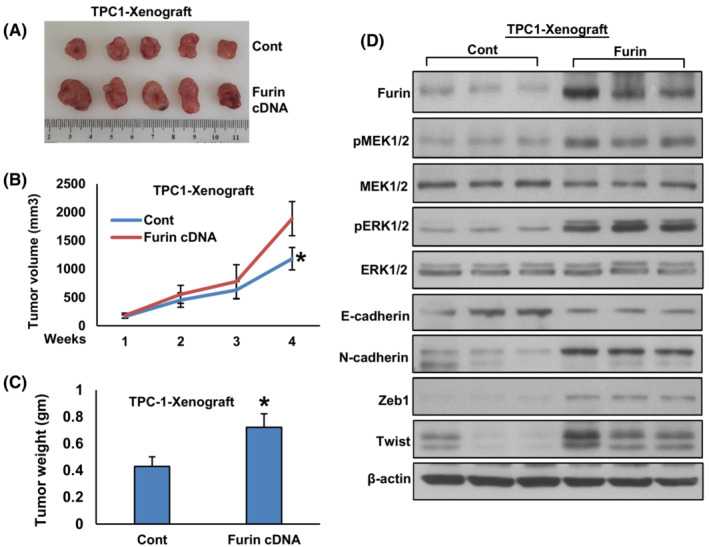
Forced expression of furin promotes tumor growth *in vivo*. (A) TPC‐1 cells were transfected with empty vector and *FURIN* cDNA, subcutaneously inoculated into the right flanks of NU/J mice (*n* = 5). The tumor volume (B) and mouse body weight were monitored weekly. After 4 weeks, mice were sacrificed and individual tumors were weighed (C), then snap‐frozen in liquid nitrogen for storage. Data were presented as mean ± SD (*n* = 5). (D) Tumors were lysed and proteins were immuno‐blotted with antibodies against Furin, pMEK1/2, MEK1/2, pERK1/2, ERK1/2, E‐cadherin, N‐cadherin, Zeb1, Twist and β‐Actin as indicated (*n* = 3). Statistical analyses were performed using two‐tailed Student's *t*‐tests. *P < 0.05.

**Fig. 11:**
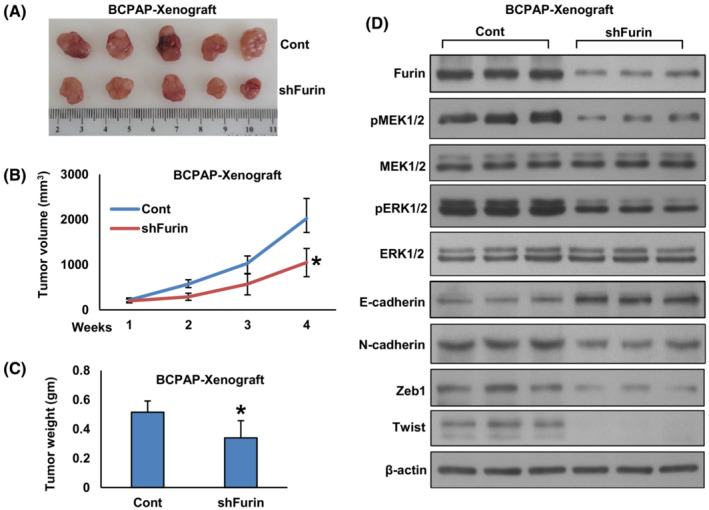
Depletion of furin delayed tumor growth *in vivo*. (A) BCPAP cells were transfected with empty vector and FURIN shRNA, subcutaneously inoculated into the right flanks of NU/J mice (*n* = 5). The tumor volume (B) and mouse body weight were monitored weekly. After 4 weeks, mice were sacrificed and individual tumors were weighed (C). Data were presented as mean ± SD (*n* = 5). (D) Tumors were lysed and proteins were immuno‐blotted with antibodies against Furin, pMEK1/2, MEK1/2, pERK1/2, ERK1/2, E‐cadherin, N‐cadherin, Zeb1, Twist and β‐Actin as indicated (*n* = 3). Statistical analyses were performed using two‐tailed Student's *t*‐tests. *P < 0.05.

We showed that furin promotes PTC growth *in vitro*. To confirm these findings *in vivo*, TPC‐1 cells were transfected with empty vector (TPC‐1‐pCMV6‐myc‐DDK) and furin cDNA (TPC‐1‐pCMV6‐myc‐DDK‐furin), subcutaneously inoculated into the right flanks of NU/J mice. After 4 weeks, the animals were sacrificed and their tumor weight was measured and proteins isolated. We found that forced expression of furin significantly increased tumor volume (Fig. 10A,B) and tumor weight (Fig. 10C) in NU/J mice. There was a noticeable increase in the expression of Furin, pMEK1/2, pERK1/2, N‐cadherin, Zeb1 and Twist with an associated downregulation of E‐cadherin expression in furin‐expressing tumors (Fig. 10D). Conversely, depletion of furin in the BCPAP cell line showed delayed tumor growth in NU/J mice as shown by a reduction in tumor volume (Fig. 11A,B) and weight (Fig. 11C). Also, downregulation in the expression of Furin, pMEK1/2, pERK1/2, N‐cadherin, Zeb1, and Twist with upregulation of E‐cadherin expression (Fig. 11D) was observed in furin‐depleted tumors. All these results indicate that furin promotes tumor growth *in vivo* and is a potential therapeutic target.

### 3.8 Depletion of Furin potentiated the antitumor effect of selumetinib *in vivo*

**Fig. 12:**
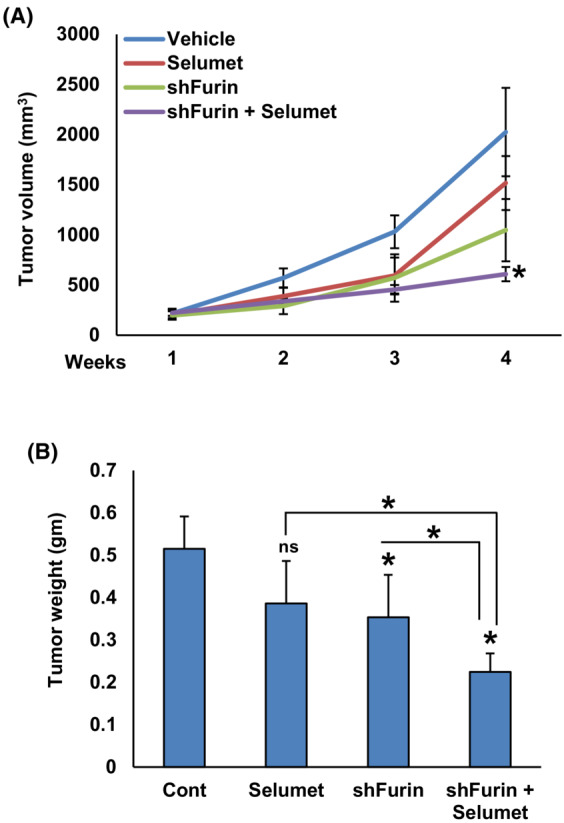
Depletion of furin potentiated the antitumor effect of selumetinib *in vivo*. NU/J mice were divided into four groups (*n* = 5) and the first two groups of mice were inoculated with BCPAP‐pRS cells (empty vector) and were treated with vehicle (0.1% DMSO, i.p) and selumetinib (10 mg·kg^−1^, i.p) respectively. The third and fourth groups of mice were inoculated with BCPAP‐pRS‐shfurin cells and treated with vehicle (0.1% DMSO, i.p) and selumetinib (10 mg·kg^−1^, i.p) respectively. After tumors grew to about 100 mm^3^, mice were treated intraperitoneally with selumetinib (10 mg·kg^−1^) and vehicle (0.1% DMSO), twice a week for 30 days. The tumor volume (A) and mouse body weight were monitored weekly. After 4 weeks, mice were sacrificed and individual tumors were weighed (B). Data were presented as mean ± SD (*n* = 5). Statistical analyses were performed using two‐tailed Student's *t*‐tests. *P < 0.05.

We showed that MEK/ERK signaling cascade has a crucial role in furin‐induced PTC cell growth and furin functions upstream to this pathway. Therefore, we wanted to investigate whether the depletion of furin potentiates the antitumor effect of selumetinib (MEK inhibitor) *in vivo*. For this experiment, NU/J mice were divided into four groups (5 mice per group) and the first two groups of mice were inoculated with BCPAP‐pRS cells (empty vector) and were treated with vehicle (0.1% DMSO, i.p) and selumetinib (10 mg·kg^−1^, i.p) respectively. The third and fourth groups of mice were inoculated with BCPAP‐pRS‐shfurin cells and treated with vehicle (0.1% DMSO, i.p) and selumetinib (10 mg·kg^−1^, i.p) respectively. After 4 weeks, the animals were sacrificed and their tumor weight was measured. We found that depletion of furin significantly potentiated the antitumor effect of selumetinib as shown by slower tumor growth (Fig. 12A) and tumor weight (Fig. 12B). However, there was no significant delay in tumor growth in the selumetinib alone treated group.

## 4 Discussion

In this study, we revealed that furin is highly expressed in 44.6% of PTC tumor samples (659/1479). The upregulation of furin is found to be significantly associated with several aggressive clinicopathological parameters such as advanced stage, tall cell variant, extrathyroidal extension, and high ATA risk. Furthermore, furin expression is also associated with *BRAF* mutation. Interestingly multivariant variant analysis shows furin expression is an independent marker for poor overall survival in PTC patients in this cohort.

We demonstrate that targeting furin impairs the malignant phenotype of the PTC cell line by activating the *BRAF* mutation. Mechanistically, *in vitro* analysis shows that overexpression of furin increased cell growth, while its knockdown in BCPAP and K1 cell lines significantly inhibited cell growth and induced apoptosis. Furthermore, ectopic expression of furin increased the invasive and migratory potential as well as activated EMT in the PTC cell line, whereas its depletion reversed the effect, which further confirms the role of furin not only in tumor growth but also promoting metastatic potential of PTC cells. These findings are consistent with previous studies that highlight the oncogenic role of furin in cancers from the ovarian [23] and lung [29].

Interestingly, our study also revealed a potential role of furin in the regulation of cancer stem cell‐like cells in PTC. Silencing of furin significantly suppressed the spheroid growth and downregulated stem cell markers such as CD44, CD133, and NANOG in PTC. In an attempt to investigate the mechanism of furin‐induced cell growth, we overexpressed furin in the TPC‐1 cell line and analyzed the expression of MEK/ERK pathway proteins. Interestingly, we find that forced expression of furin markedly increased the phosphorylation of MEK1/2 and ERK1/2 in that cell line, while the opposite effect is seen with stable knockdown of furin.

Our data also demonstrate that furin functions as upstream to MEK/ERK signaling cascade by threatening *BRAF* mutated cell lines with a pharmacological inhibitor of MEK, mirdametinib, and selumetinib. These inhibitors suppress the activation of MEK1/2 and ERK1/2 with no effect on furin expression. We also show that co‐inhibition of furin and MEK prominently reduced cell proliferation, migration, invasion, EMT, spheroid growth, and stemness in PTC cells, showing the role of MEK/ERK signaling pathway in furin‐mediated cancer stemness maintenance.

The oncogenic role of furin in PTC cell is also suggested by *in vivo* analysis where forced expression of furin significantly increase tumor growth in NU/J mice, while its depletion showed the opposite effect. Since *in vitro* knockdown of *FURIN* also inhibited activation of the MEK/ERK signaling cascade which plays an important role in PTC. Therefore, we studied whether depletion of furin could potentiate the antitumor effect of selumetinib (MEK inhibitor) *in vivo*, and indeed we were able to see the synergistic antitumor effect of furin depletion and selumetinib in NU/J mice.

The previous report has shown that furin has many additional substrates that might contribute to the observed oncogenic effect of many cancers like pancreatic cancer [19] and colorectal cancer [26]. So, it would not be surprising that our results show the synergistic effect of *FURIN* knockdown and inhibition of MEK oncogenic pathway in PTC. This synergistic strategy could potentially enhance therapeutic efficacy in treating aggressive or metastatic PTC. Since data from this study and previous studies indicate that furin inhibition has a promising antitumor effect and can simultaneously inhibit multiple oncogenic pathways in several cancers.

Our studies have several limitations, the expression of Furin has been studied retrospectively on a single institute cohort from specific ethnicity. Additional studies are needed to determine the extent of furin overexpression in PTC tumor samples.

## 5 Conclusion

Our study shows that furin is upregulated in clinical PTC samples and it could be used as a prognostic marker for poor survival. *In vitro* and *in vivo* analysis demonstrates that furin inhibition can be used as a therapeutic strategy in PTC.

## Author contributions

PKP and AKS: Designed, performed experiments, and wrote the manuscript. DP: Performed experiments and data analysis. SKP: Prepared the TMA and conducted all the immunohistochemistry experiments and scoring of IHC spots. RD, ST, RB, and WH: Performed experiments. FHA: Technical help for animal experiments. SSA and FA: Contributed samples and analyzed clinical data. KSA: Designed, implemented the study, wrote, and critically reviewed the manuscript. This is to confirm that all authors read and approved the final manuscript.

## Conflict of interest

The authors declare no conflict of interest.

### Peer review

The peer review history for this article is available at https://publons.com/publon/10.1002/1878‐0261.13396.

## Supporting information


**Fig. S1.** Decrease in cell growth after furin depletion was due to apoptosis.

## Data Availability

All data generated or analyzed during this study are included in this published article.
