# Supplementary material for: Overexpression of the pro‐protein convertase furin predicts prognosis and promotes papillary thyroid carcinoma progression and metastasis through RAF/MEK signaling
Source: Mol Oncol. 2023 Feb 27;17(7):1324–42. doi: 10.1002/1878-0261.13396 (PMC10323895; doi:10.1002/1878-0261.13396)
Supplement: Supplementary file 1 — Fig. S1. Decrease in cell growth after furin depletion was due to apoptosis. [file MOL2-17-1324-s001.zip › Suppl Figure 1 Legend.docx]

**Supplementary Figure 1: Decrease in cell growth after furin depletion was due to apoptosis. (A)** PTC cells were pre-treated with zVAD-fmk (80 µM) for three hours followed by transfection with *FURIN* siRNA. After 48 hours, cells were stained with fluorescein-conjugated annexin-V and propidium iodide (PI) and analyzed by flow cytometry. Data were presented as mean ± SD (n = 3). **(B)** PTC cells after zVAD-fmk pre-treatment and *FURIN* siRNA transfection, clonogenic assay was performed. Data were presented as mean ± SD (n = 3). **(C)** PTC cells were transfected with scrambled siRNA and two different *FURIN* siRNA sequences (25 nM). After 48 hours, cells were stained with PI followed by flow cytometry analysis. Data were presented as mean ± SD (n = 3). Statistical analyses were performed using two-tailed Student's t-tests. *p < 0.05.
