# Supplementary figures and images for: Overexpression of the pro‐protein convertase furin predicts prognosis and promotes papillary thyroid carcinoma progression and metastasis through RAF/MEK signaling
Source: Mol Oncol. 2023 Feb 27;17(7):1324–42. doi: 10.1002/1878-0261.13396 (PMC10323895; doi:10.1002/1878-0261.13396)

Supplementary Figure 1

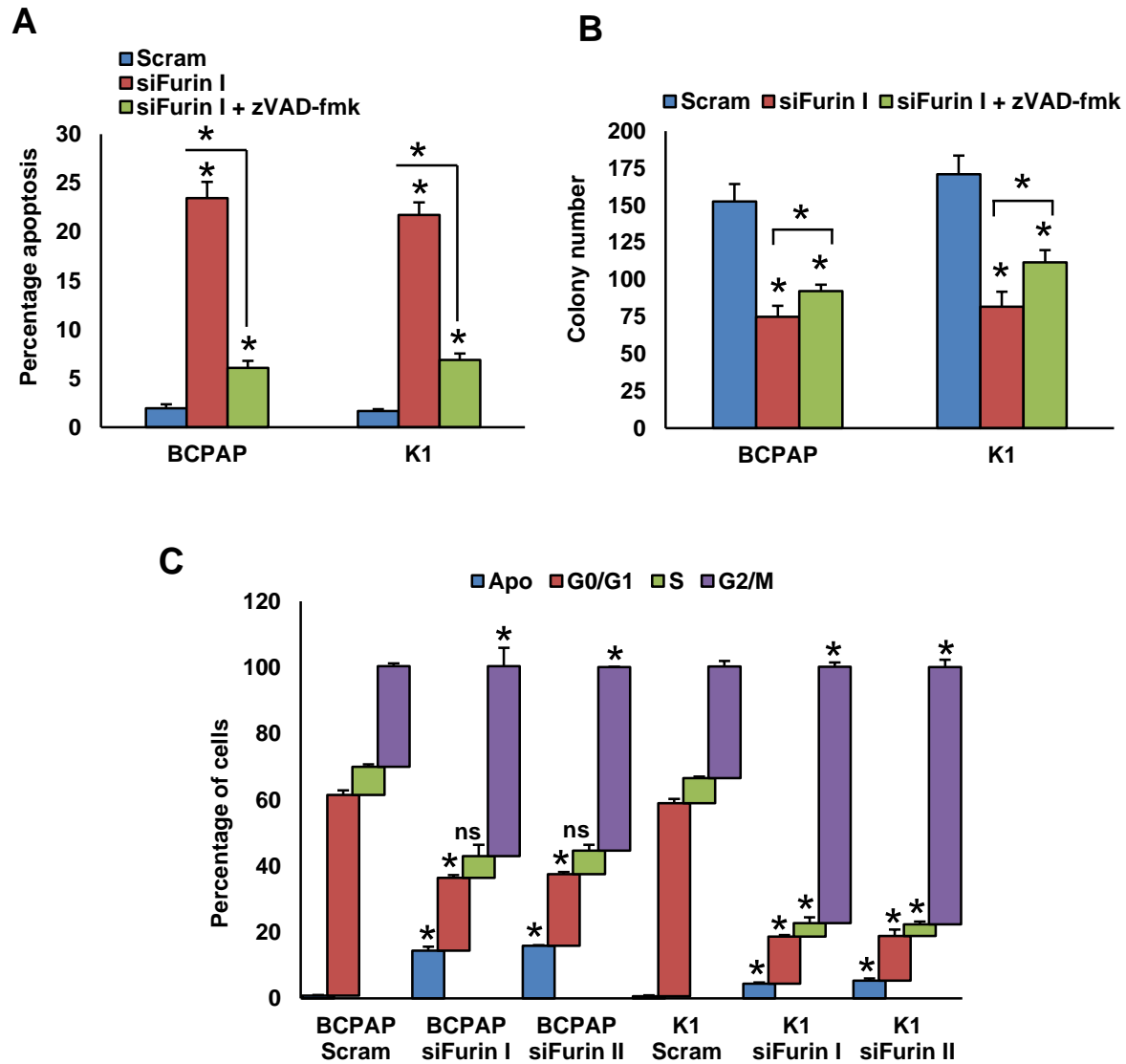

Supplement: Supplementary file 1 — Fig. S1. Decrease in cell growth after furin depletion was due to apoptosis. [file MOL2-17-1324-s001.zip › Suppl Figure 1.pdf]
